# Classification of the Occurrence of Dyslipidemia Based on Gut Bacteria Related to Barley Intake

**DOI:** 10.3389/fnut.2022.812469

**Published:** 2022-03-24

**Authors:** Satoko Maruyama, Tsubasa Matsuoka, Koji Hosomi, Jonguk Park, Mao Nishimura, Haruka Murakami, Kana Konishi, Motohiko Miyachi, Hitoshi Kawashima, Kenji Mizuguchi, Toshiki Kobayashi, Tadao Ooka, Zentaro Yamagata, Jun Kunisawa

**Affiliations:** ^1^Research and Development Department, Hakubaku Co., Ltd., Yamanashi, Japan; ^2^Laboratory of Vaccine Materials, Center for Vaccine and Adjuvant Research and Laboratory of Gut Environmental System, National Institutes of Biomedical Innovation, Health and Nutrition, Osaka, Japan; ^3^Department of Health Sciences, School of Medicine, University of Yamanashi, Yamanashi, Japan; ^4^Artificial Intelligence Center for Health and Biomedical Research, National Institutes of Biomedical Innovation, Health and Nutrition, Osaka, Japan; ^5^Department of Physical Activity Research, National Institutes of Biomedical Innovation, Health and Nutrition, Tokyo, Japan; ^6^Laboratory of Computational Biology, Institute for Protein Research, Osaka University, Osaka, Japan; ^7^Department of Microbiology and Immunology, Kobe University Graduate School of Medicine, Hyogo, Japan; ^8^Graduate Schools of Medicine, Graduate School of Pharmaceutical Sciences, Graduate Schools of Science, Graduate School of Dentistry, Osaka University, Osaka, Japan; ^9^International Research and Development Center for Mucosal Vaccines, The Institute of Medical Science, The University of Tokyo, Tokyo, Japan; ^10^Research Organization for Nano and Life Innovation, Waseda University, Tokyo, Japan

**Keywords:** barley, dyslipidemia, gut bacteria, responder, machine learning

## Abstract

Barley is a grain rich in β-glucan, a soluble dietary fiber, and its consumption can help maintain good health and reduce the risk of metabolic disorders, such as dyslipidemia. However, the effect of barley intake on the risk of dyslipidemia has been found to vary among individuals. Differences in gut bacteria among individuals may be a determining factor since dietary fiber is metabolized by gut bacteria and then converted into short-chain fatty acids with physiological functions that reduce the risk of dyslipidemia. This study examined whether gut bacteria explained individual differences in the effects of barley intake on dyslipidemia using data from a cross-sectional study. In this study, participants with high barley intake and no dyslipidemia were labeled as “responders” to the reduced risk of dyslipidemia based on their barley intake and their gut bacteria. The results of the 16S rRNA gene sequencing showed that the fecal samples of responders (*n* = 22) were richer in *Bifidobacterium, Faecalibacterium, Ruminococcus* 1, *Subdoligranulum*, Ruminococcaceae UCG-013, and *Lachnospira* than those of non-responders (*n* = 43), who had high barley intake but symptoms of dyslipidemia. These results indicate the presence of certain gut bacteria that define barley responders. Therefore, we attempted to generate a gut bacteria-based responder classification model through machine learning using random forest. The area under the curve value of the classification model in estimating the effect of barley on the occurrence of dyslipidemia in the host was 0.792 and the Matthews correlation coefficient was 0.56. Our findings connect gut bacteria to individual differences in the effects of barley on lipid metabolism, which could assist in developing personalized dietary strategies.

## Introduction

Dyslipidemia, a disorder in lipid metabolism characterized by high levels of LDL-cholesterol and/or triglycerides and low HDL-cholesterol levels, is a well-established risk factor for cardiovascular disease (1, 2). According to the World Health Organization, one-third of ischemic heart diseases worldwide is attributable to high cholesterol, with elevated cholesterol being estimated to cause 2.6 million deaths (4.5% overall) and 29.7 million disability-adjusted life years (3). In Japan, the government estimated 2,205,000 dyslipidemia patients (4), representing a public health problem.

The causes of dyslipidemia include genetics (5) and dietary habits, such as excessive dietary lipid intake (6). In addition to dietary interventions limiting fat and carbohydrates intake, other ways of treating dyslipidemia may benefit from the food ingested. The active intake of resistant starch and dietary fiber has been proposed as an example diet that effectively improves dyslipidemia (7). A double-blind, randomized crossover trial reported that corn-resistant starch improved lipid metabolism by increasing insulin sensitivity in obese men (8). In addition, several meta-analyses have shown that barley β-glucan reduced serum LDL-cholesterol (9, 10). However, individual differences in the effects of foods on host energy sensitivity have been identified, which has not been resolved for functional foods (11, 12). One reason for these individual differences is thought to be due to differences in the composition of gut bacteria among humans (13).

In addition to the physical properties of the dietary fiber we consume, such as inhibiting lipid and carbohydrate absorption, there are physiological effects of the metabolites converted by gut bacteria (14). These metabolites are short-chain fatty acids (SCFAs), such as acetate, propionate, and butyrate (15), which are highly bioactive and exhibit physiological functions, such as improving lipid metabolism, insulin sensitivity, and oxidative stress (16, 17). Since different gut microbiomes cause differences in the production of metabolites, including SCFAs, the function of foods might depend on an individual's gut microbiome composition. For example, the soluble fiber β-glucan in barley is a crucial nutrient source for gut bacteria (18, 19); therefore, differences in the gut microbiome composition can lead to differences in the health effects of β-glucan. Kovatcheva-Datchary et al. (20) observed that the effects of barley on postprandial blood glucose levels for some subjects (responders) improved glucose metabolism after barley intake, while for others (non-responders), it did not. Moreover, they mentioned that responders had a higher ratio of *Prevotella/Bacteroides* (i.e., the two dominant gut bacteria) than non-responders. Since *Prevotella* has been reported to promote glycogen storage in the liver and improve host glucose metabolism, it has been suggested that *Prevotella* is involved in improving glucose metabolism by barley in the gut (20).

Individual differences in barley's effects on the occurrence of dyslipidemia may result from these differences in gut bacteria. However, differences in gut bacteria by race and place of residence are known to exist (21), and there have been no detailed studies on the Japanese population. Identifying the gut bacteria specific to barley in dyslipidemia could improve the field's understanding of the relationship between barley and lipid metabolism as well as the mechanisms of this association. This would allow for the predictions of individual differences in the effects of barley, such as barley responder determination based on gut bacteria. Further, understanding these differences could provide insights for developing individualized dietary programs for disease prevention in healthy populations. Therefore, this study aimed to determine whether gut bacteria could explain individual differences in the effect of barley on the occurrence of dyslipidemia.

The data from a cross-sectional study of Japanese adults (*N* = 130) were analyzed; participants were divided into two groups by barley intake—“responders” without dyslipidemia and “non-responders” with dyslipidemia. We compared the gut bacteria in both groups and examined whether these bacteria explained the effects of barley by identifying the gut bacterial characteristics of responders. Finally, we generated a barley responder classification model based on gut bacteria using machine learning to predict barley responders.

## Materials and Methods

### Study Design and Participants

The current study used the first year of data of the participants over age 40 who were enrolled in a larger project, “The cohort study on barley and intestinal environment (UMIN000033479).” The objective of the overall study, UMIN000033479, was to assess the associations between the health impact of barley and gut bacteria. The study was conducted in accordance with the principles of the Declaration of Helsinki. The sampling occurred from August 2018 to March 2019. The current analyses in this study examined the gut bacterial characteristics of barley responders with dyslipidemia.

**Figure 1:**
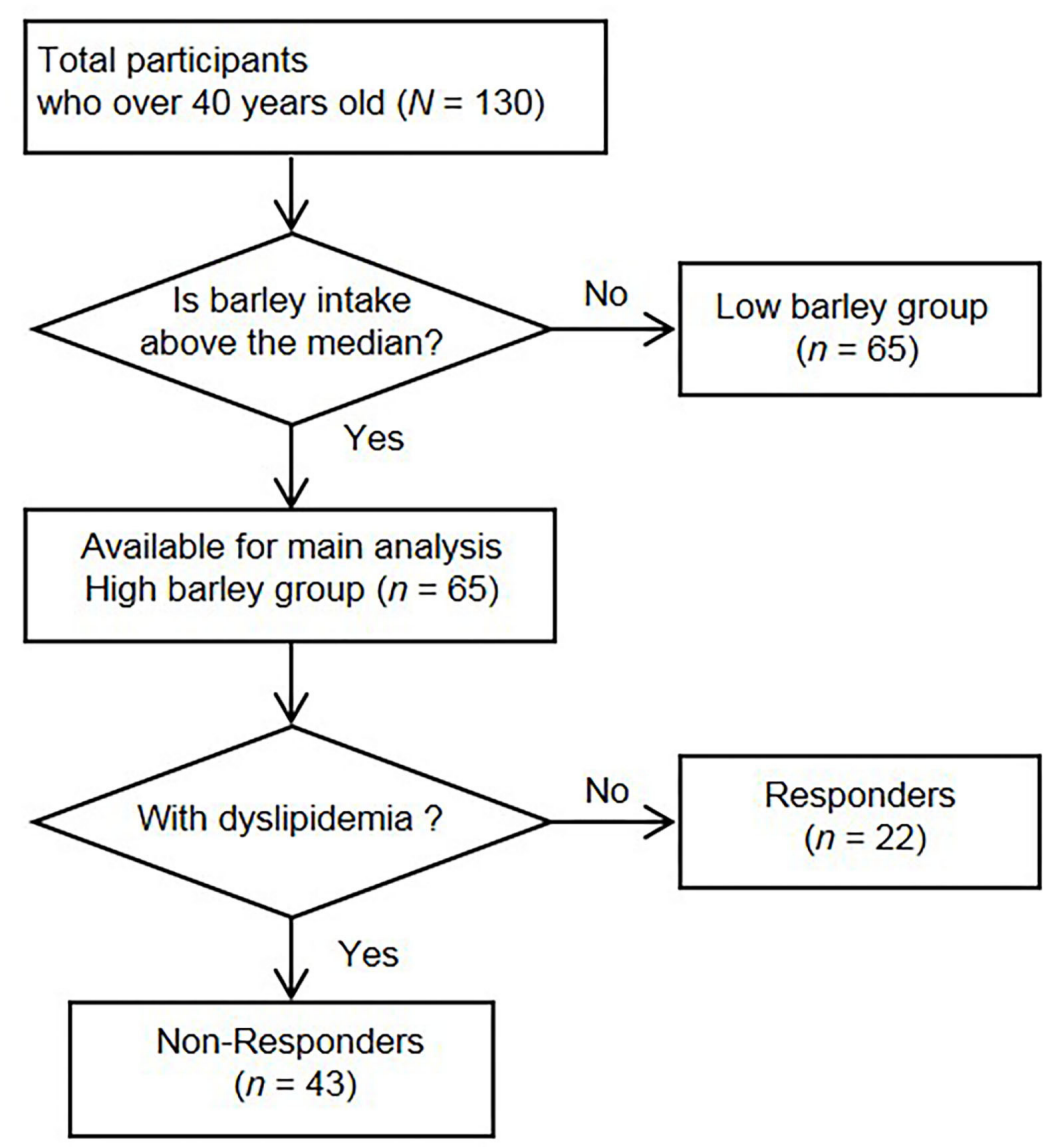
Flow chart of the recruitment and selection of participants.

In the original study, 272 participants provided informed consent, and 236 participants had complete data. In the current analyses, we excluded 106 participants under age 40 years from the final analyses because they had a lower risk of dyslipidemia. The remaining 130 participants were classified into either a “high barley or “low barley” group based on their median barley consumption rate (g/1,000 kcal). The high barley group was the primary analytic group for this study. Figure 1 shows the flowchart of the sample in this study.

### Measures

We assessed the daily total energy (kcal/day) of participants using a brief self-administered diet history questionnaire (BDHQ, Gender Medical Research, Inc., Tokyo, Japan). Daily barley intake (g/day) was calculated using a questionnaire. Specifically, we asked about the size of the rice bowl (large-size 200 g, middle-size 160 g, small-size 140 g, child-size 100 g), the proportion of barley to white rice in the bowl (none, 5, 10, 15, 30, 50%), number of bowls per day (number), and frequency of consumption per month (none, 0.5, 1, 4, 8, 16, or more days/month). Then, we calculated the daily barley consumption rate (g/1,000 kcal) for each participant using their daily total energy and barley intake. We asked the participants to submit a copy of their health examination reports to study their basic information, such as body weight and blood test. Moreover, details including sex, age, history of diseases, medications, smoking habits, daily activity, evacuation condition, and fermented food and supplement consumption habits were collected using the questionnaire.

### DNA Extraction and 16S rRNA Gene Amplicon Sequencing

Fecal samples were collected in containers of guanidine thiocyanate (GuSCN) solution at home. DNA was extracted from fecal samples and stored at room temperature for up to 30 days following previously described methods (21). Briefly, 0.2 mL of fecal sample in GuSCN solution was mixed with 0.3 mL of lysis buffer (No. 10, Kurabo Industries Ltd., Osaka, Japan) and 0.5 g of 0.1 mm glass beads (WakenBtech Co., Ltd., Tokyo, Japan). It was homogenized using a Cell Destroyer PS1000 (Bio Medical Science, Tokyo, Japan) at 4,260 rpm for 50 sec at room temperature. The homogenized sample was centrifuged at 13,000 × g for 5 min at room temperature, and DNA was extracted from the supernatant using a Gene Prep Star PI-80X device (Kurabo Industries Ltd). The concentration of the extracted DNA was determined using a NanoDrop Spectrophotometer ND-1000 (Thermo Fisher Scientific Inc., DE, USA). The samples were stored at −30°C until use. 16S rRNA gene amplification and sequencing were performed following previously published procedures (22). Barcoded amplicons were generated from the V3–V4 region of the 16S rRNA gene from the fecal DNA samples using the following primers: forward: 5-TCGTCGGCAGCGTCAGATGTGTATAAGCGACAGCCTACGGGNGGCWGCAG-3; reverse, 5-GTCTCGTGGGCTCGGAGATGTGTATAAGAGACAGGACTACHVGGGTATCTAATCC-3′. The Nextera XT Index Kit v2 Set A (Illumina Inc., CA, USA) was used to prepare the DNA library for Illumina MiSeq. The concentration of the DNA library was determined using the QuantiFluor dsDNA System (Promega, Co., MI, USA), and 16S rRNA gene sequencing was performed using Illumina MiSeq (Illumina) in accordance with the manufacturer's instructions.

### Bioinformatics Analysis

The Quantitative Insights Into Microbial Ecology (QIIME) software package (version 1.9.1) (23) was used to analyze the sequence reads from the Illumina MiSeq. The steps from the trimming of paired-end reads to an operational taxonomic unit (OTU) picking were performed automatically using QIIME Analysis Automating Script (Auto-q) (24). OTU picking was performed based on sequence similarity (>97%) using open-reference OTU picking with UCLUST software against the SILVA v128 reference sequence. The taxonomy (phylum, class, order, family, genus) and relative abundance were calculated using the SILVA v128 database (25, 26). We created a rarefaction curve (Supplementary Figure 1) by using vegan R-package (version 2.5-7) to confirm the relationship between the number of sequence reads and the measured OTUs. Based on this curve, we randomly selected 10,000 reads per sample for statistical analysis; this read depth was enough to observe true species richness.

### Selection of Responders and Non-responders

We stratified the patients into the dyslipidemia group (including mild abnormality) and the non-dyslipidemia group based on blood lipids from health examinations and medication status. All data were up-to-date, reported within 1 year, and there were no participants with missing data. Participants were categorized as being in the dyslipidemia group if they had at least one of the following: (1) triglycerides of 150 mg/dL or higher, (2) HDL-cholesterol of <40 mg/dL, (3) LDL-cholesterol of 120 mg/dL or higher, or (4) taking lipid metabolism-related medication. Those who did not fit any of the criteria were assigned to the non-dyslipidemia group.

From the high barley group, we identified the group of responders (*n* = 22) who were in the non-dyslipidemia group. The remaining participants in the high barley group were categorized as non-responders (*n* = 43). In brief, barley responders were defined as participants in the high barley group without problems related to lipid metabolism, while barley non-responders were participants who had lipid metabolism problems despite being in the high barley group.

### Data Analysis

All data analyses were conducted in R version 3.6.0 (27).

#### Statistical Analyses

α-diversity (within-subject species diversity) indices of Chao 1, Shannon, and Simpson were calculated using the estimated richness function of the phyloseq R-package (version 1.30.0). We performed Student's independent *t*-tests on age, body mass index, blood pressure, fasting blood glucose, hemoglobin A1c, triglyceride, LDL-cholesterol, and HDL-cholesterol between responders and non-responders. We performed the Mann–Whitney *U* test to determine differences in α-diversity and the abundance of gut bacteria. As a sensitivity analysis, we additionally performed the analysis of composition of microbiomes (ANCOM) by using nlme R-package (version 3.1-155) and compositions R-package (version 2.1.3). We set α =.05 at 70% of the comparisons in ANCOM, with the W statistics corresponding to the number of times the abundance of the gut bacteria differed significantly among the subject groups (28). The compared gut bacteria were based on the 64 families and top 50 genera that were sorted by mean relative abundance in all subjects over 40 years old. All *p*-values derived from the difference in α-diversity, medical check-up, and microbiota were corrected for multiple testing by the method of false discovery rate (FDR) and then called *q*-values. In this study, the gut bacteria with *p* < 0.05 were considered significant.

#### Principal Coordinate Analysis of Microbiomes at the Genus Level

We summarized the composition of the gut microbiome by principal coordinate analysis (PCoA) using the vegdist function of the vegan R-package (version 2.5-7), the quasieuclid function, and the dudi.pco function of the ade4 R-package (version 1.7-16) to generate figures. Data were calculated using the Bray-Curtis distance.

#### Random Forest Machine Learning

This study used supervised classification learning of random forest models to predict dyslipidemia as responders or non-responders. Sixty-five participants in the high barley group were used as the dataset. Forty-seven (70%) were randomly selected as the training set to train the model, with the remaining being used to evaluate the model's performance. The bacteria of the top 50 genera were used as the variables. The classification model was generated using the RandomForest R-package (version 6.6-14) and the caret R-package (version 6.0-86). We also generated a baseline model by the RandomForest R-package using default values for all parameters. Next, repeated cross-validation was performed to address the small sample size and improve the evaluation of the model. The number of folds was 13, and the number of repetitions was 10. For hyperparameters, the ntree was set to 500, and mtry was tuned using the caret R-package. The other parameters used default values. The importance of each variable was calculated using the varImp function of the caret R-package. The model was evaluated using a receiver operating characteristic (ROC) curve (ROCR R-package, version 1.0-11). To measure the relative performance, we calculated the area under the ROC curve (AUC) and the Matthews correlation coefficient (MCC) (29).

## Results

### Participant Characteristics

The participants' mean age was 51 years (*SD* = 6), with a range of 40–65 years. Of the total participants, 104 (80%) were males, and the remaining 26 (20%) were females. The median barley intake of the 130 participants was 3.68 (interquartile range: 1.17, 8.49) (g/1,000 kcal), and participants were stratified into a high barley group (*n* = 65) and a low barley group (*n* = 65) based on this value (Figure 1). Participants meeting at least one criterion were defined as non-responders who had dyslipidemia, and the remaining non-dyslipidemia subjects were defined as responders. Twenty-two were responders, and 43 were non-responders (Figure 1 and Table 1). No significant differences in age and sex were found between the two groups. In terms of health indicators, systolic blood pressure [116 (17), 127 (17), *p* = 0.026] and diastolic blood pressure [74 (13), 84 (11), *p* = 0.004] were lower in responders, while the mean values for non-responders were categorized as borderline, although they were not medically hypertensive. Fasting blood glucose [88 (7), 97 (15), *p* = 0.002] and hemoglobin A1c levels [5.4 (0.2), 5.6 (0.4), *p* = *0*.014] were also lower in responders than in non-responders; however, the non-responders mean values did not meet the diagnostic threshold for diabetes nor were they in the borderline category. Thus, non-responders had no major abnormalities other than dyslipidemia, but responders generally had a good health status, including low blood pressure and blood glucose levels along with normal lipid metabolism markers.

**Table 1:** Characteristics of the study participants of each group.

	**Overall (*N* = 130)**	**Responders (*n* = 22)**	**Non-responders (*n* = 43)**	
	***mean1*** **(*SD*) or *n* (%)**	***mean*** **(*SD*) or *n* (%)**	***mean*** **(*SD*) or *n* (%)**	***p-*** **value^a^**
Male (*n*, %)	104 (80%)	15 (68%)	37 (86%)	0.17^b^
Age (years)	51 (6)	48 (6)	51 (7)	0.07
Weight (kg)	67.1 (12.3)	59.8 (11.2)	72.3 (10.7)	<0.001
BMI (kg/m^2^)	23.4 (3.7)	21.1 (3.0)	25.0 (3.1)	<0.001
Systolic blood pressure (mmHg)	122 (16)	116 (17)	127 (17)	0.03
Diastolic blood pressure (mmHg)	79 (12)	74 (13)	84 (11)	0.003
Fasting blood glucose (mg/dL)	95 (12)	88 (7)	97 (15)	0.002
Hemoglobin A1c (%)	5.6 (0.4)	5.4 (0.2)	5.6 (0.4)	0.014
Triglyceride (mg/dL)	125 (86)	61 (24)	159 (89)	<0.001
HDL-cholesterol (mg/dL)	60 (16)	71 (15)	54 (16)	<0.001
LDL-cholesterol (mg/dL)	123 (29)	95 (12)	136 (25)	<0.001

*BMI, body mass index; SD, standard deviation*.

a *Compared responders and non-responders using Student's independent t-tests except for sex*.

b *Compared responders and non-responders using Pearson's chi-squared test*.

### Gut Bacteria Specific to Barley Responders

**Figure 2:**
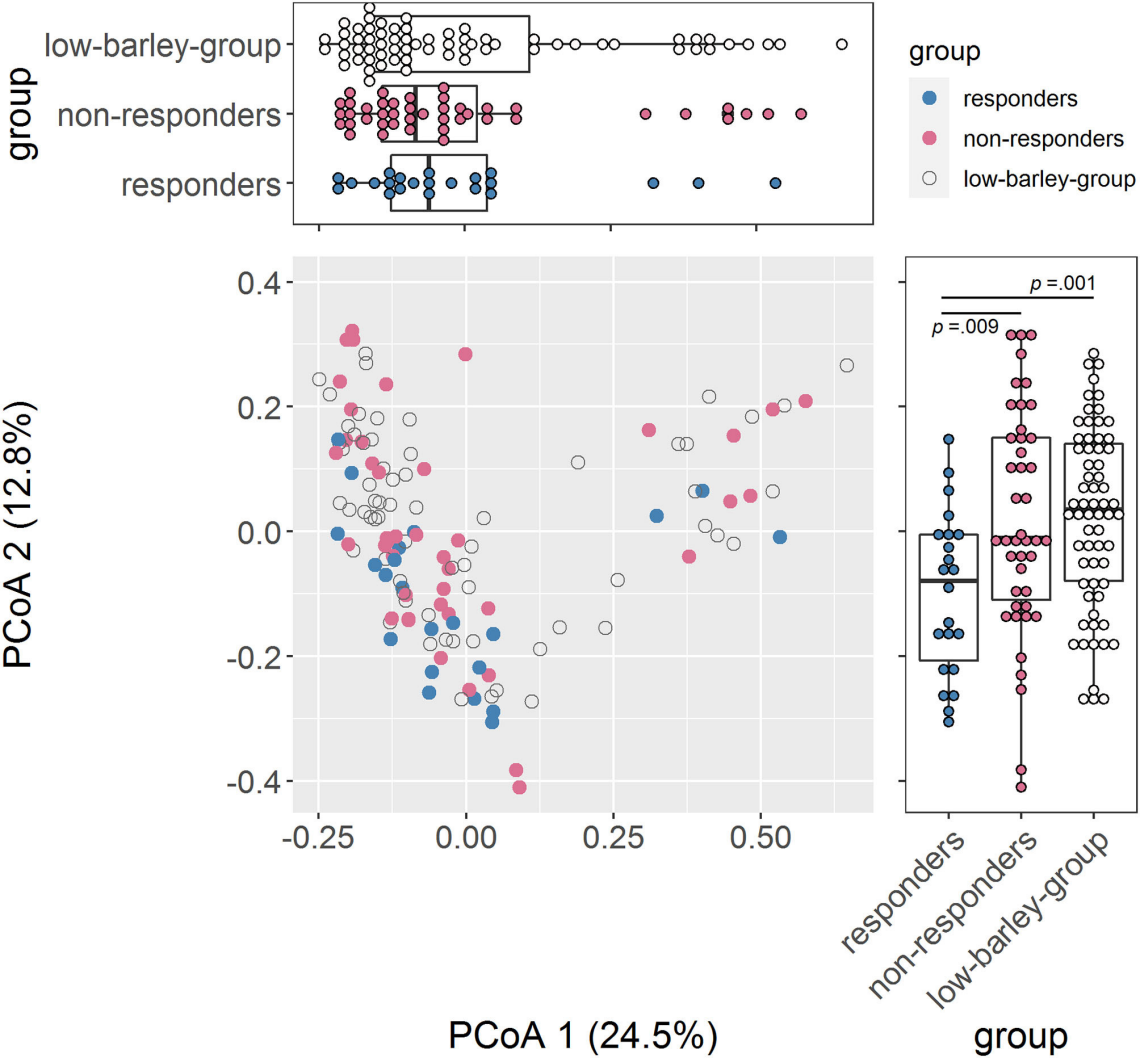
Comparison of the gut microbiome composition. PCoA of gut microbiome based on 266 genera abundance.

We compared gut bacteria between responders and non-responders to examine the involvement of gut bacteria in the risk of dyslipidemia due to barley intake. First, we compared α-diversity, which was significantly higher in Chao1 and tended to be higher in Shannon for responders than for non-responders, indicating that responders had a higher diversity of gut microbiome (Table 2). Next, we compared the distribution of the gut microbiome of all participants by PCoA and found a significant difference between responders and non-responders in PCoA2 (*p* = 0.009; Figure 2). In PCoA2, significant differences between the responders and low barley groups were observed (*p* = 0.001), but there was no difference between the non-responders and low barley groups (*p* = 0.90; Figure 2), indicating that the gut microbiome of responders was specific.

**Table 2:** α-diversity of each group.

	**Overall (*N* = 130)**	**Responders (*n* = 22)**	**Non-responders (*n* = 43)**	
	***Median*** **(interquartile range)**	***Median*** **(interquartile range)**	***Median*** **(interquartile range)**	***p-*** **value^a^**
Chao1	1,077 (887, 1,263)	1,237 (968, 1404)	951 (824, 1,107)	0.009
Shannon	3.69 (3.35, 3.96)	3.81 (3.58, 3.94)	3.48 (3.23, 3.80)	0.07
Simpson	0.94 (0.90, 0.95)	0.94 (0.90, 0.95)	0.92 (0.90, 0.95)	0.20

a *Compared responders and non-responders using Mann–Whitney U-test*.

**Figure 3:**
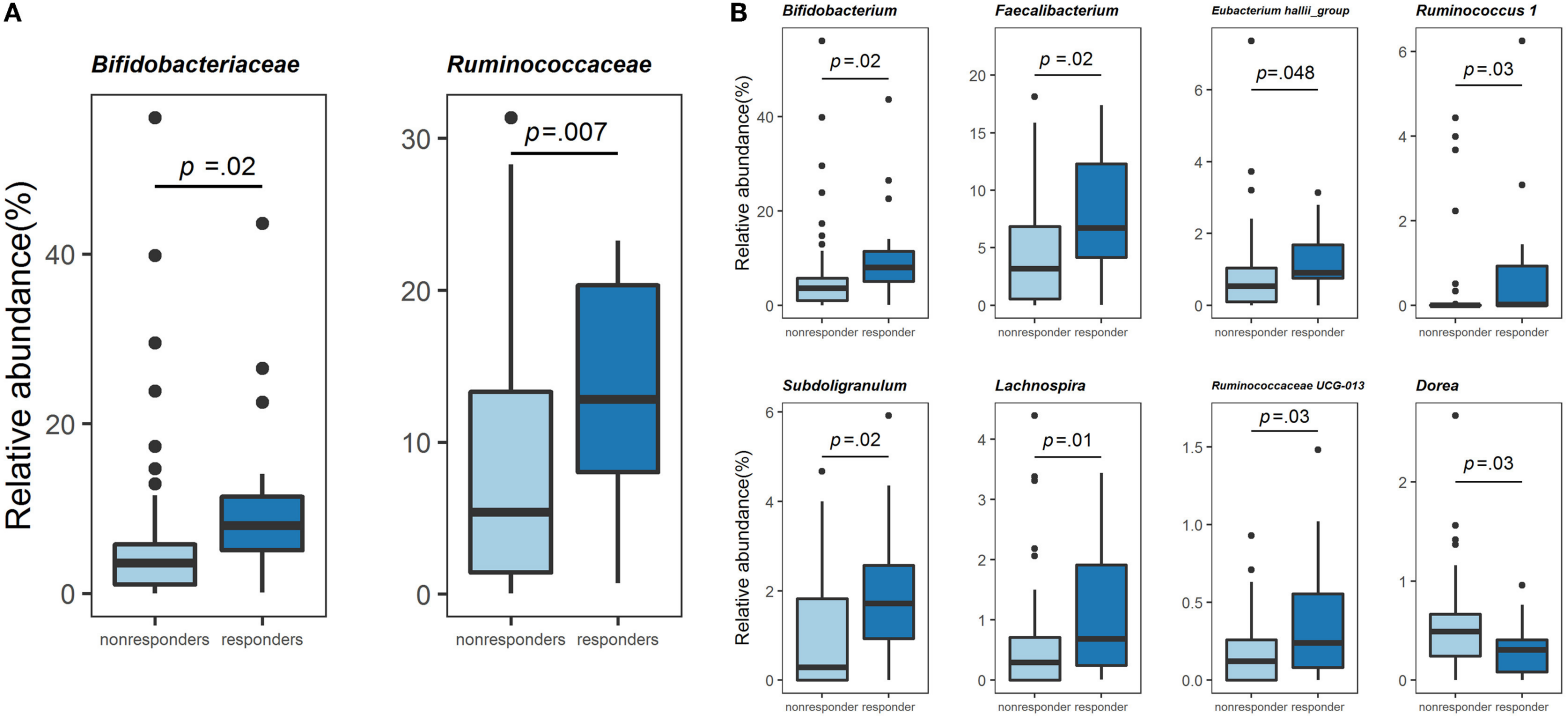
Comparison of the gut microbiome composition. **(A)** Relative abundance (%) of the two families specific to responders. **(B)** Relative abundance (%) of the eight genera specific to responders.

Next, we compared the gut bacterial composition of responders and non-responders. At the family level, Bifidobacteriaceae [7.96 (5.06, 11.41), 3.58 (1.02, 5.77), *p* = 0.02] and Ruminococcaceae [6.73 (3.86, 11.23), 2.19 (0.56, 6.61), *p* = 0.007] were significantly higher in responders than in non-responders (Mann-Whitney *U*-test, Figure 3A). These two families were also significantly high using ANCOM (Supplementary Figure 2A). At the genus level, relative abundance comparison among the top 50 genera revealed the most minor bacteria had an average relative abundance of 0.213%. Results of comparison using the Mann–Whitney *U*-test revealed that *Bifidobacterium* [7.96 (5.06, 11.41), 3.58 (1.02, 5.77), *p* = 0.02], *Faecalibacterium* [6.73 (3.86, 11.23), 2.19 (0.56, 6.61), *p* = 0.02], *Eubacterium hallii* group Conversely, further analysis using ANCOM showed that only *Bifidobacterium* was significantly different at the genus level. However, the gut bacteria with non-significant but high W statistics were generally reproducible in analysis using the Mann-Whitney *U*-test (Supplementary Figure 2B). Thus, responders were a specific population in terms of gut bacterial composition, suggesting that the physiological effects of these gut bacteria might be related to lipid metabolism in the host. Among the gut bacteria that changed in both high and low barley groups, *Bifidobacterium* and *Subdoligranulum* were significantly more abundant in the non-dyslipidemia than in the dyslipidemia groups, even in the low barley group (Supplementary Table 1). This suggests that these two genera may contribute to the reduction of dyslipidemia, regardless of barley intake. Therefore, the six genera characterized only in the high barley group (i.e., *Faecalibacterium, Eubacterium hallii_*group*, Ruminococcus* 1, Ruminococcaceae UCG-013*, Lachnospira*, and *Dorea*) may be involved in the interaction with barley to improve dyslipidemia.

### Gut Bacteria-Based Classification of Barley Responder

**Figure 4:**
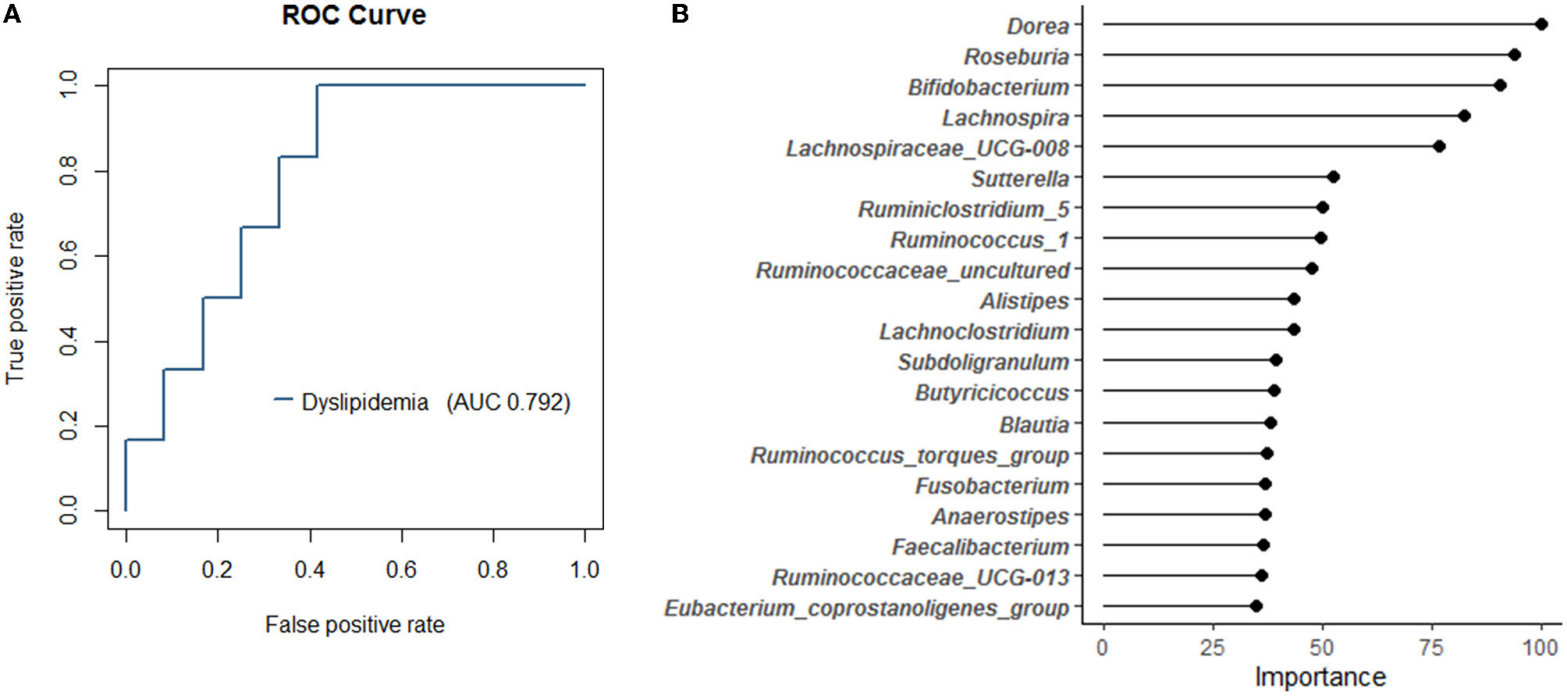
The random forest classification model generated based on 50 genera in the training data set. **(A)** The receiver operating characteristic (ROC) curves and area under curve (AUC) of the microbiome for discrimination between responders and non-responders. **(B)** The top 20 explanatory variables that are important for the classification model.

Since a marked difference in the relative abundance of gut bacteria in the top 50 genera between responders and non-responders was observed, we attempted to predict responders using a random forest model with these 50 genera. We accurately determined the AUC of the classification model using repeated cross-validation. As a result, we created a responder classification model with an AUC of 0.792 for the test set (Figure 4A). The optimal cutoff value for this model was 0.324, with a sensitivity of 1.0 and a specificity of 0.583, resulting in MCC of 0.56. The AUC of the training set was 1.0. The baseline model created as a reference had MCC of 0.13. Thus, setting the appropriate parameters and performing repeated cross-validation could improve the resolution of the model. This model suggests that gut bacterial composition could be involved as a determinant of host responsiveness to dyslipidemia. The top 20 gut bacteria that were essential explanatory variables for this classification model included *Dorea, Bifidobacterium*, and *Faecalibacterium* (Figure 4B). The relative abundance of these bacteria differed between responders and non-responders (Figure 3); therefore, this model reflects the taxonomic characteristics of responders and may facilitate future clinical interventions to manage lipid metabolism.

## Discussion

This study included a relatively large sample size from a population with a high barley intake. In addition, we created a more versatile classification model using the top 50 genera in relative abundance as the explanatory variables. We also used repeated cross-validation when developing the model, which increases its classification ability. We used these methods to test whether gut bacteria contributed to individual differences in lipid metabolic responses to barley intake in a sample of Japanese adults over age 40. To the best of our knowledge, this is the first study linking individual differences in the effects of barley on lipid metabolism to gut bacteria. While barley intake improves lipid metabolism, its effects are not always consistent (30). In this study, participants with high barley intake did not necessarily have low levels of dyslipidemia. Our analytic strategy allowed us to identify the gut bacteria that define reactivity. Furthermore, we constructed a model to predict the reactors based on gut bacteria, which showed that the health-promoting effect of barley on dyslipidemia could be stratified according to gut bacteria.

We compared the relative abundance of 50 genera by the Mann-Whitney *U*-test and found that responders were enriched in *Bifidobacterium, Faecalibacterium, Eubacterium hallii* group*, Ruminococcus* 1, *Subdoligranulum*, Ruminococcaceae UCG-013, and *Lachnospira* and deficient in *Dorea* compared to non-responders. Therefore, these eight genera are characteristic gut bacteria of barley responders with dyslipidemia. Besides, in the sensitivity analysis using ANCOM, *Bifidobacterium* was also significantly abundant in responders, and the other gut bacteria characteristic of responders also generally had high W statistics; these further results increase the robustness of our results. In addition, the enrichment of *Bifidobacterium* and *Subdoligranulum* observed in the non-dyslipidemia group compared to the dyslipidemia group of the low barley group, as shown by the Mann-Whitney *U*-test, could be a marker for distinguishing between healthy and unhealthy individuals, regardless of barley intake. *Bifidobacterium* is well known to utilize various carbohydrates (31, 32). Previous studies have shown that barley β-glucan (33) and polysaccharides, such as galacto-oligosaccharides (34), arabino-oligosaccharides (35), and inulin (36) can be widely utilized as energy sources, with acetic acid as the primary metabolite. Several studies with humans and animals investigating the effects of prebiotics reported that *Bifidobacterium* and acetic acid increased in the feces or cecum as a result of prebiotics (37, 38). Therefore, it seems reasonable that the gut without dyslipidemia was enriched in *Bifidobacterium*, regardless of barley intake in this study. Although the energy source and mechanism of *Subdoligranulum* and its involvement in dyslipidemia are unclear, it is known to produce butyrate, which improves lipid metabolism (39, 40). A clinical study in Taiwan and China has reported that *Subdoligranulum* is depleted in patients with inflammatory bowel disease, suggesting that it may be a helpful bacteria (41, 42).

The other six genera differed only in the high barley group, suggesting their involvement in the barley-dependent improvement of dyslipidemia. One mechanism by which fiber-rich foods, such as barley, improves lipid metabolism is through SCFAs produced via gut bacterial metabolism. SCFAs have been shown to affect host energy metabolism by activating various G-protein coupled receptors (43). For example, in mouse studies, SCFAs were found to activate G-protein coupled receptor 43 (GPR43) (44). GPR43 is expressed in adipose, intestinal, and immune tissues and promotes the secretion of leptin, which breaks down fat and inhibits lipid uptake by adipose tissue (43). These findings suggest that gut bacteria fermentation influences host lipid metabolism via “energy harvesting,” the synthesis of SCFAs from barley. Consistent with these findings, the five genera that increased in the responders of this study are known to produce SCFAs. For example, *Faecalibacterium, Ruminococcus* 1, and Ruminococcaceae UCG-013 belong to the Ruminococcaceae family, and many bacteria in this family are known to be general butyrate-producing bacteria (45–47). Although details of the metabolic pathways of *Ruminococcus* 1 and Ruminococcaceae UCG-013 have not been reported, *Faecalibacterium prausnitzii*, the only species belonging to *Faecalibacterium*, has been reported to have the ability to ferment complex carbohydrates (48, 49). In addition, *F. prausnitzii* can take up acetic acid produced by bacteria, such as *Bifidobacterium*, to produce butyric acid (50). Therefore, the finding that responders had enriched *Faecalibacterium* could be related to the fermentation of barley β-glucan and conversion to butyrate.

*Eubacterium hallii* is a well-known butyrate-producing bacterium like those belonging to Ruminococcaceae. However, unlike *F. prausnitzii*, it cannot utilize complex carbohydrates. Previous studies have reported that the energy source of *E. hallii* is monosaccharides or intermediate metabolites [acetate or lactate (48, 51)]. Thus, the *Eubacterium hallii_*group may not utilize barley directly and instead uses intermediate metabolites from other bacteria that can utilize barley to produce butyrate. Therefore, we analyzed the correlation between *Eubacterium hallii_*group and other bacteria in the responders; however, it did not correlate with well-known acetic acid-producing bacteria, such as *Bifidobacterium* (Supplementary Table 2). *Eubacterium hallii*_group in the responder was positively correlated with *Feacalibacterium*, a responder-specific bacterium (results not shown), suggesting that these bacteria grow in the same gut environment since both are acetic acid-utilizing bacteria. Although the details of these gut bacterial communities are unclear, our findings indicate that bacteria directly utilizing barley and bacteria affecting dyslipidemia through their coexistence with other bacteria may define the responsiveness to barley in dyslipidemia.

In this study, we developed a classification model for responders based on gut bacteria using Random Forest. Generally, individual differences among study subjects are often eliminated by using multivariate analysis in epidemiological studies. However, in this study, we focused on these individual differences and found that gut bacteria can partially explain the effects of barley. If this classification model is applied in practical use, the classification ability may need further improvement. Nevertheless, the results helped interpret the importance of gut bacteria to the host. In particular, the fact that similar bacteria were found through the other analyses, such as the Mann–Whitney *U* test and ANCOM, might have increased the confidence in this classification model.

This study has several limitations. First, population characteristics, such as race and dietary habits, that differ from the current sample may affect the model's classification performance. Second, the gut microbiome composition in this study was not measured by shotgun sequencing, nor was it measured in terms of metabolites, and thus the discussion of this study is limited. Finally, this was a cross-sectional study; thus, we could not establish a causal relationship between gut bacteria and dyslipidemia. Future studies should verify the performance of our classification model in participants without bias and replicate the study in other populations with different races and lifestyles to identify possible confounding factors. In addition, we plan to conduct a long-term longitudinal study to investigate the effect of gut bacteria that are characteristic of responders on lipid metabolism.

In summary, this study showed that barley responders in dyslipidemia had distinct bacteria profiles. The possibility of stratifying a host's energy response to its diet in terms of gut bacteria in this study provides insights into the “individual differences in effects,” which has been a problem in functional studies of various foods. We performed additional machine learning studies to create a classification model that could determine the compatibility between barley and host-based gut bacteria, which could contribute to developing personalized diet strategies in the future.

## Data Availability Statement

The original contributions presented in the study are included in the article/Supplementary Material, further inquiries can be directed to the corresponding author.

## Ethics Statement

The studies involving human participants were reviewed and approved by Yamanashi University Ethics Committee (Approval No. 1824), the National Institutes of Biomedical Innovation Health and Nutrition Ethics Committee (Approval No. 169-04), and the Chiyoda Paramedical Care Clinic Ethics Committee (Approval No. 15000088). The patients/participants provided their written informed consent to participate in this study.

## Author Contributions

TM, KH, HM, TK, TO, JK, and ZY: conceptualization. TM, KH, HM, TK, MN, SM, and KK: project administration. HM, KK, MM, KH, JP, HK, KM, and JK: resources. SM and TM: investigation and writing original draft. SM, TM, and JK: writing, revising, and editing. All authors read and approved the final manuscript.

## Funding

This study was supported by funding from Hakubaku Co., Ltd., the Japan Agency for Medical Research and Development (AMED), Grant Number JP20gm1010006h004, and the Ministry of Health and Welfare of Japan and Public/Private R&D In-vestment Strategic Expansion PrograM: PRISM, Grant Number 20AC5004.

## Conflict of Interest

The department of ZY has received research grants for other studies from Hakubaku Co., Ltd. SM, TM, MN, and TK are employees of Hakubaku Co., Ltd. The authors declare that this study received funding from Hakubaku Co., Ltd. The funder had the following involvement in the study: conceptualization, project administration, investigation, writing original draft, revising, and editing.

## Publisher's Note

All claims expressed in this article are solely those of the authors and do not necessarily represent those of their affiliated organizations, or those of the publisher, the editors and the reviewers. Any product that may be evaluated in this article, or claim that may be made by its manufacturer, is not guaranteed or endorsed by the publisher.
