# Supplementary material for: Classification of the Occurrence of Dyslipidemia Based on Gut Bacteria Related to Barley Intake
Source: Front Nutr. 2022 Mar 24;9:812469. doi: 10.3389/fnut.2022.812469 (PMC8988889; doi:10.3389/fnut.2022.812469)
Supplement: Supplementary file 2 [file Image_1.PDF]

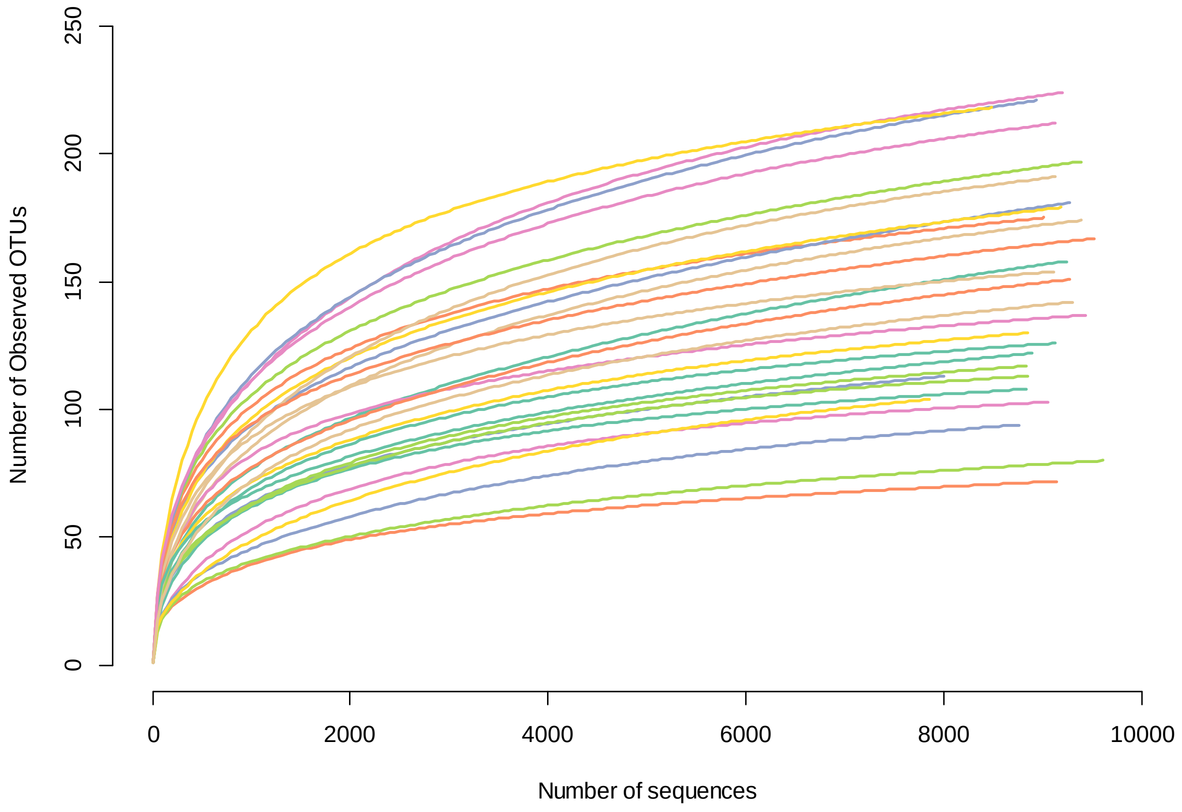

Supplementary Figure 1. The rarefaction curve to confirm the number of OTUs obtained from 16S rRNA gene amplicon sequences. The figure shows the results in 28 randomly selected subjects out of all participants (*N*=130).

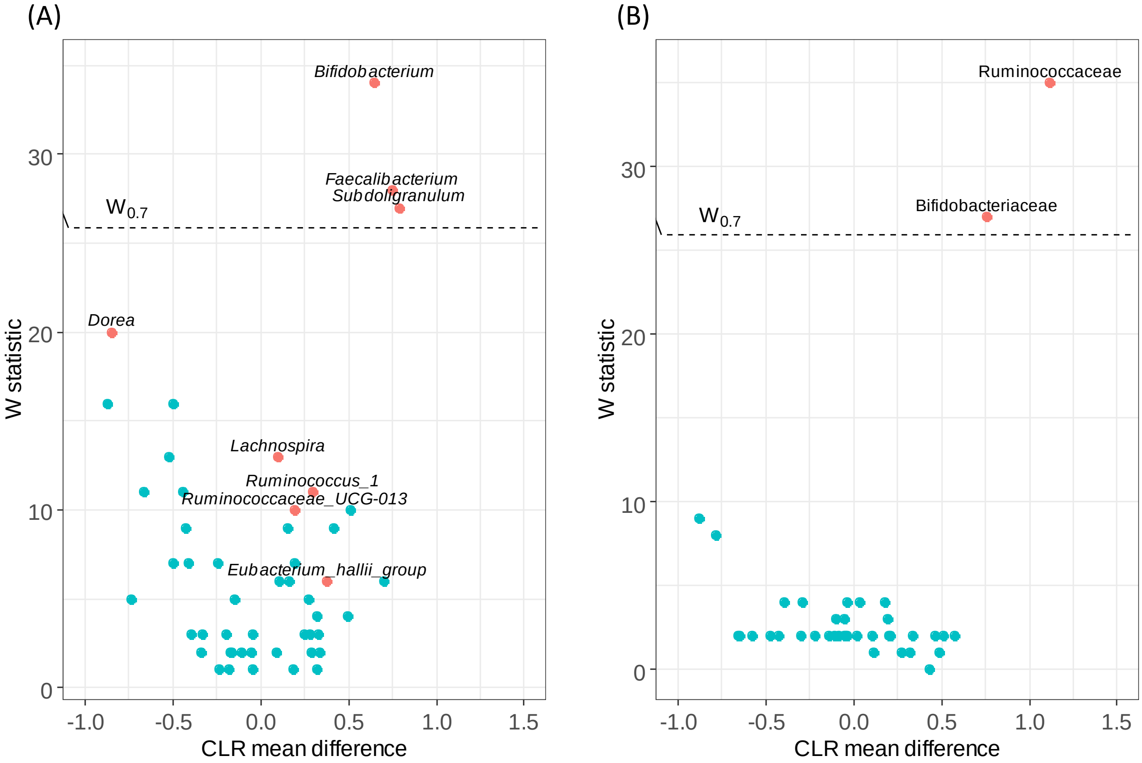

Supplementary Figure 2. The volcano plot shows the results of ANCOM. The dashed line is the set cutoff value, indicating that there was a significant difference between 70% of the subjects. Pink dots are characteristic gut bacteria of responders determined by the Mann-Whitney *U* test. **(A)** family level **(B)** genus level.
